# Conversion of spikers to bursters in pituitary cell networks: Is it better to disperse for maximum exposure or circle the wagons?

**DOI:** 10.1371/journal.pcbi.1011811

**Published:** 2024-01-30

**Authors:** Mehran Fazli, Richard Bertram

**Affiliations:** 1 Department of Mathematics, Florida State University, Tallahassee, Florida, United States of America; 2 Programs in Neuroscience and Molecular Biophysics, Florida State University, Tallahassee, Florida, United States of America; École Normale Supérieure, College de France, CNRS, FRANCE

## Abstract

The endocrine cells of the pituitary gland are electrically active, and *in vivo* they form small networks where the bidirectional cell-cell coupling is through gap junctions. Numerous studies of dispersed pituitary cells have shown that typical behaviors are tonic spiking and bursting, the latter being more effective at evoking secretion. In this article, we use mathematical modeling to examine the dynamics of small networks of spiking and bursting pituitary cells. We demonstrate that intrinsic bursting cells are capable of converting intrinsic spikers into bursters, and perform a fast/slow analysis to show why this occurs. We then demonstrate the sensitivity of network dynamics to the placement of bursting cells within the network, and demonstrate strategies that are most effective at maximizing secretion from the population of cells. This study provides insights into the *in vivo* behavior of cells such as the stress-hormone-secreting pituitary corticotrophs that are switched from spiking to bursting by hypothalamic neurohormones. While much is known about the electrical properties of these cells when isolated from the pituitary, how they behave when part of an electrically coupled network has been largely unstudied.

## Introduction

The pituitary is the body’s master endocrine gland. It contains five types of endocrine cells: somatotrophs secrete growth hormone, lactotrophs secrete the milk-producing hormone prolactin, corticotrophs secrete the stress hormone adrenocorticotropic hormone, gonadotrophs secrete the reproductive hormones follicle-stimulating hormone and luteinizing hormone, and thyrotrophs secrete thyroid stimulating hormone. Each of these cell types is electrically active, generating electrical impulses or bursts of impulses, which are longer events [1]. The electrical events are driven by the opening of Ca^2+^ channels in the cell plasma membrane, which both depolarize the membrane and bring Ca^2+^ into the cell. Exocytosis of hormone-filled vesicles is then evoked by the binding of Ca^2+^ to fusion proteins, releasing the hormone into the blood stream. Thus, elevation of the intracellular Ca^2+^ concentration is the main driver of hormone secretion, although other factors are involved. Evidence from the Stojilkovic lab has established that bursts of impulses are likely more effective at releasing hormone than spikes not clustered into bursts, since the intracellular Ca^2+^ level rises much higher in the former than in the latter because the events are longer, allowing Ca^2+^ to accumulate to a higher level [2]. This hypothesis was later supported by results from a modeling study demonstrating the effectiveness at evoking secretion of Ca^2+^ fluctuations resulting from bursts of impulses [3].

For many years, there was little appreciation of a network structure of endocrine pituitary cells, and virtually all *in vitro* studies were performed on isolated cells [1]. However, publications from the Mollard lab in the early 2000s changed that viewpoint. Studying slices of the pituitary, fluorescent techniques established that these cells are arranged into populations of networks situated adjacent to fenestrated capillaries that allow hypothalamic neurohormones to reach the pituitary cells and that allow hormone released by the pituitary cells to enter the general circulation [4–7]. Each small network appears to consist of cells of the same type, and there are many such networks. Electrophysiological and morphological methods established that the cells within a network are connected through gap junction proteins that electrically couple them together [7]. The networks are plastic; the coupling between cells changes in response to stimulating factors and according to the physiological state of the animal. This has been characterized both morphologically and through Ca^2+^ imaging, which shows a much higher degree of coordination among lactotrophs from female mice during lactation than prior to lactation [8], and similar high coordination among somatotrophs in response to growth hormone releasing hormone [9]. These findings were represented using functional network graphs, where each node represents a cell and each edge indicates that Ca^2+^ time courses of two cells are largely coordinated (independent of whether the cells are actually connected by gap junctions). In the pituitaries from lactating mice, for example, the network is much denser, and the degree distribution of nodes in the graph satisfies a power law, and is thus a scale-free functional network [8]. Whether the structural network, where the edges represent gap junctions, is also scale-free is not known. In a recent study, we showed that the relationship between the structural network of pituitary-type bursters and the resulting functional network is tenuous at best, at least in the case of weak coupling that we considered [10]. For example, the hubs of the functional network do not correspond to the hubs of the structural network.

In that prior study, we examined networks of electrically-coupled endocrine pituitary cells in which all cells were identical and produced a bursting pattern when uncoupled. Yet, there is a great deal of heterogeneity in the electrical activity of pituitary cells, with some cells tonically spiking, some bursting, and some silent [11]. In stress-hormone-releasing corticotrophs, the neurohormonal signal from the hypothalamus that evokes hormone secretion switches the cells from tonic spiking to bursting [12, 13]. In general, spiking cells can be thought of as non-secreters since they secrete little hormone, while those that are bursting are actively secreting hormone [2]. In pituitary somatotrophs, lactotrophs, and corticotrophs, the bursts typically have very small spikes riding on a depolarized plateau, a form of bursting often referred to as “pseudo-plateau bursting” [14]. This is more effective at secreting hormone than spiking since it has a longer duration [2, 3].

In this study, we use mathematical modeling to examine the functional network properties of structural networks consisting of heterogeneous collections of electrically coupled endocrine pituitary cells, some spiking and some bursting. We start with small networks of just two model lactotroph cells, and then move to larger networks of up to 20 cells, which is a reasonable number for pituitary networks (the anterior pituitary contains many such small networks). We focus on three questions. First, if a spiker and a burster are coupled together, is one able to convert the other to its type of activity, and if so, how does it happen? Second, how sensitive is the network activity to a small change in the intrinsic activity of a cell within that network? Third, since bursting is the cell state that evokes hormone secretion, what is the best strategy for arranging the bursting cells within a network so as to maximize secretion?

## The mathematical model

The mathematical model characterizing electrical activity in pituitary lactotrophs that we use [15] has low dimensionality. It has variables for the membrane potential (*V*), the free cytosolic Ca^2+^ concentration (*c*), and activation variables *n* and *b* for the delayed rectifier K^+^ and big conductance K^+^ channels (known as BK channels). The variables change in time according to:

$$\begin{array}{c} C_{m}\frac{dV}{dt}=-\left(I_{\text{Kdr}}+I_{\text{Ca}}+I_{\text{BK}}+I_{\text{SK}}+I_{\text{L}}+I_{\text{c}}\right), \end{array}$$ (1)

$$\begin{array}{c} \tau_{n}\frac{dn}{dt}=n_{\infty }\left(V\right)-n, \end{array}$$ (2)

$$\begin{array}{c} \frac{dc}{dt}=-f_{c}\left(\alpha I_{\text{Ca}}+k_{c}c\right), \end{array}$$ (3)

$$\begin{array}{c} \tau_{b}\frac{db}{dt}=b_{\infty }\left(V\right)-b. \end{array}$$ (4)

The ionic currents, *I*_Kdr_ (delayed rectifier K^+^ current), *I*_Ca_ (L-type Ca^2+^ current), *I*_BK_ (big conductance K^+^ current), *I*_SK_ (small conductance K^+^ current), and *I*_L_ (leak current), are generated by the flux of ions through open ion channels between the cytosol and extracellular spaces. The electrical coupling current, denoted by *I*_c_, is due to ion fluxes between adjacent cells via gap junctions. The parameter *C*_*m*_ is the membrane capacitance, *f*_*c*_ is the fraction of cytosolic Ca^2+^ that is free, *k*_*c*_ is the pumping rate through Ca^2+^ pumps in the plasma membrane, and *α* is a conversion factor from Ca^2+^ current to concentration. Time constants for K^+^ channel activation are *τ*_*n*_ and *τ*_*b*_.

**Fig 1:**
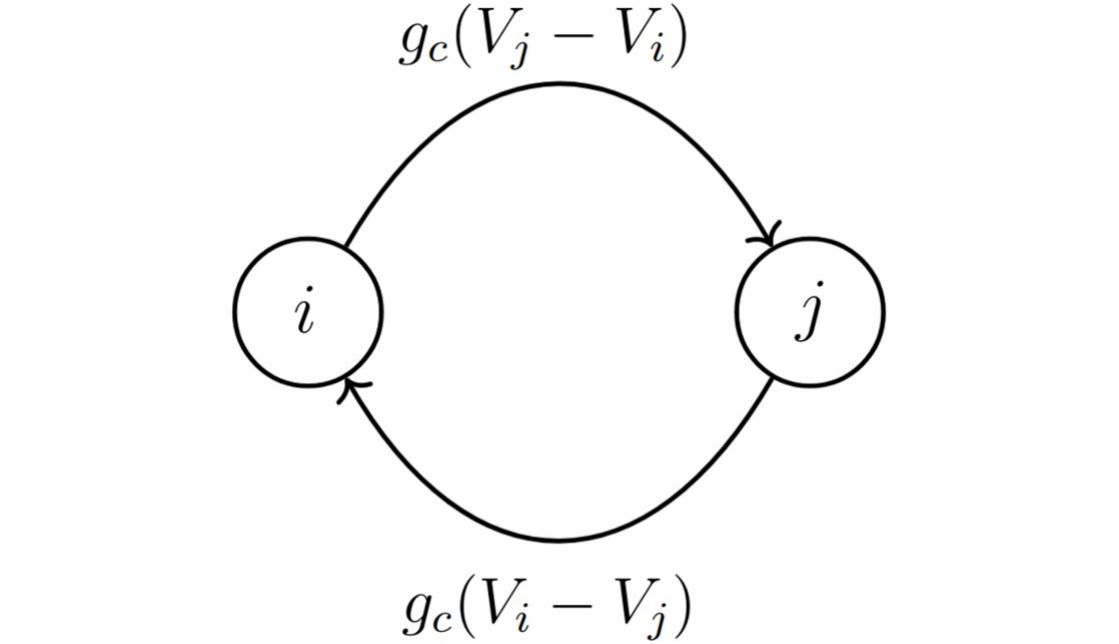
Bidirectional electrical coupling. The modeling of gap junction between two pituitary lactotrophs, denoted as cells *i* and *j*. The coupling conductance is *g*_*c*_ and the magnitude of the coupling current is *g*_*c*_|*V*_*i*_ − *V*_*j*_|.

The mathematical expressions for ionic currents are:

$$\begin{array}{c} I_{\text{Kdr}}=g_{\text{Kdr}}n\left(V-V_{\text{K}}\right), \end{array}$$ (5)

$$\begin{array}{c} I_{\text{Ca}}=g_{\text{Ca}}m_{\infty }\left(V\right)\left(V-V_{\text{Ca}}\right), \end{array}$$ (6)

$$\begin{array}{c} I_{\text{BK}}=g_{\text{BK}}b\left(V-V_{\text{K}}\right), \end{array}$$ (7)

$$\begin{array}{c} I_{\text{SK}}=g_{\text{SK}}d_{\infty }\left(c\right)\left(V-V_{\text{K}}\right), \end{array}$$ (8)

$$\begin{array}{c} I_{\text{L}}=g_{\text{L}}\left(V-V_{\text{L}}\right), \end{array}$$ (9)

$$\begin{array}{c} I_{\text{c}}=\sum_{j\in N_{i}}g_{c}\left(V_{i}-V_{j}\right), \end{array}$$ (10)

where *g*_*x*_ refers to the maximal conductance for *x* ∈ {Kdr, Ca, BK, SK, L} and *g*_*c*_ is the electrical coupling conductance due to gap junctions. Additionally, *V*_*x*_ (for *x* ∈ {K, Ca, L}) is the Nernst potential for each corresponding current. For the coupling current *I*_c_, *V*_*i*_ and *V*_*j*_ correspond to the cells connected through gap junctions. As depicted in Fig 1, the coupling current into cell *i* from any cell *j* is *g*_*c*_(*V*_*i*_ − *V*_*j*_), while that into cell *j* has the same magnitude but opposite sign: *g*_*c*_(*V*_*j*_ − *V*_*i*_).

Equilibrium functions for gating variables, denoted as *x*_∞_(*V*) (for *x* ∈ {*n*, *b*, *m*}), are defined by:

$$\begin{array}{c} x_{\infty }\left(V\right)=\frac{1}{1+\text{exp}(\frac{\nu_{x}-V}{l_{x}})}, \end{array}$$ (11)

where *ν*_*x*_ and *l*_*x*_ refer to the half-activation membrane voltage and the slope factor for the function, respectively. The gating variable *m* is assumed to be at a quasi-equilibrium state, so *m* = *m*_∞_(*V*). For the SK channel activation variable *d*, which is governed by the cytosolic Ca^2+^ concentration:

$$\begin{array}{c} d_{\infty }=\frac{c^{2}}{c^{2}+k_{\text{SK}}^{2}}. \end{array}$$ (12)

In Eq (10), $N_{i}$ represents the set of electrically-connected neighbors of cell *i*. The dimensionless secretion value is calculated using an increasing sigmoid function:

$$\begin{array}{c} s=\frac{1}{1+\text{exp}(-5(\frac{c-0.27}{0.082}-0.6))}. \end{array}$$ (13)

Parameter values in this function were set so that *s* would increase substantially during each burst.

To study interactions between bursters and spikers in a network, we utilize the random walk algorithm to build the network of pituitary endocrine cells. The algorithm starts with two connected nodes. Then in a loop, we randomly choose a node in the current network then, with probability *p*, we add a new node to the network by connecting it to the chosen node. Otherwise, we randomly connect the chosen node to a node already in the network. This process is repeated until we have a network with *n* nodes [16].

The parameters used in the model and their corresponding values are provided in Table 1. Using these parameter values, the model produces a pseudo-plateau bursting pattern. To produce a tonic spiking pattern, the BK conductance is set to 0. The differential equations were solved using a 4th-order Runge-Kutta method in Python with time step of Δ*t* = 0.5 ms. The code can be accessed at www.math.fsu.edu/~bertram/software/pituitary.

**Table 1:** Parameter values for an intrinsic burster. Intrinsic spikers have *g*_BK_ = 0 nS.

Name	Value	Name	Value
*C*_*m*_	5 pF	*ν*_*n*_	−5 mV
*g*_Kdr_	2.5 nS	*ν*_*m*_	−20 mV
*g*_Ca_	2.1 nS	*ν*_*b*_	−5 mV
*g*_L_	0.2 nS	*l*_*n*_	10 mV
*g*_SK_	2 nS	*l*_*m*_	12 mV
*g*_BK_	1 nS	*l*_*b*_	2 mV
*g*_*c*_	0.002 nS	*α*	0.0015 *μ*M/fC
*V*_Ca_	60 mV	*f*_*c*_	0.005
*V*_K_	−75 mV	*k*_*c*_	0.12 *μ*M
*V*_L_	−50 mV	*k*_SK_	0.4 *μ*M
*τ*_*n*_	30 ms	*τ*_*b*_	5 ms

## Studies of two coupled cells

In this section, we investigate the behavior of a small system in which a tonic spiking cell is electrically coupled to a pseudo-plateau bursting cell. We first report on computer simulations using different coupling strengths, and then perform a fast/slow analysis to explain the results.

### A spiker is converted to a burster with sufficiently strong coupling

**Fig 2:**
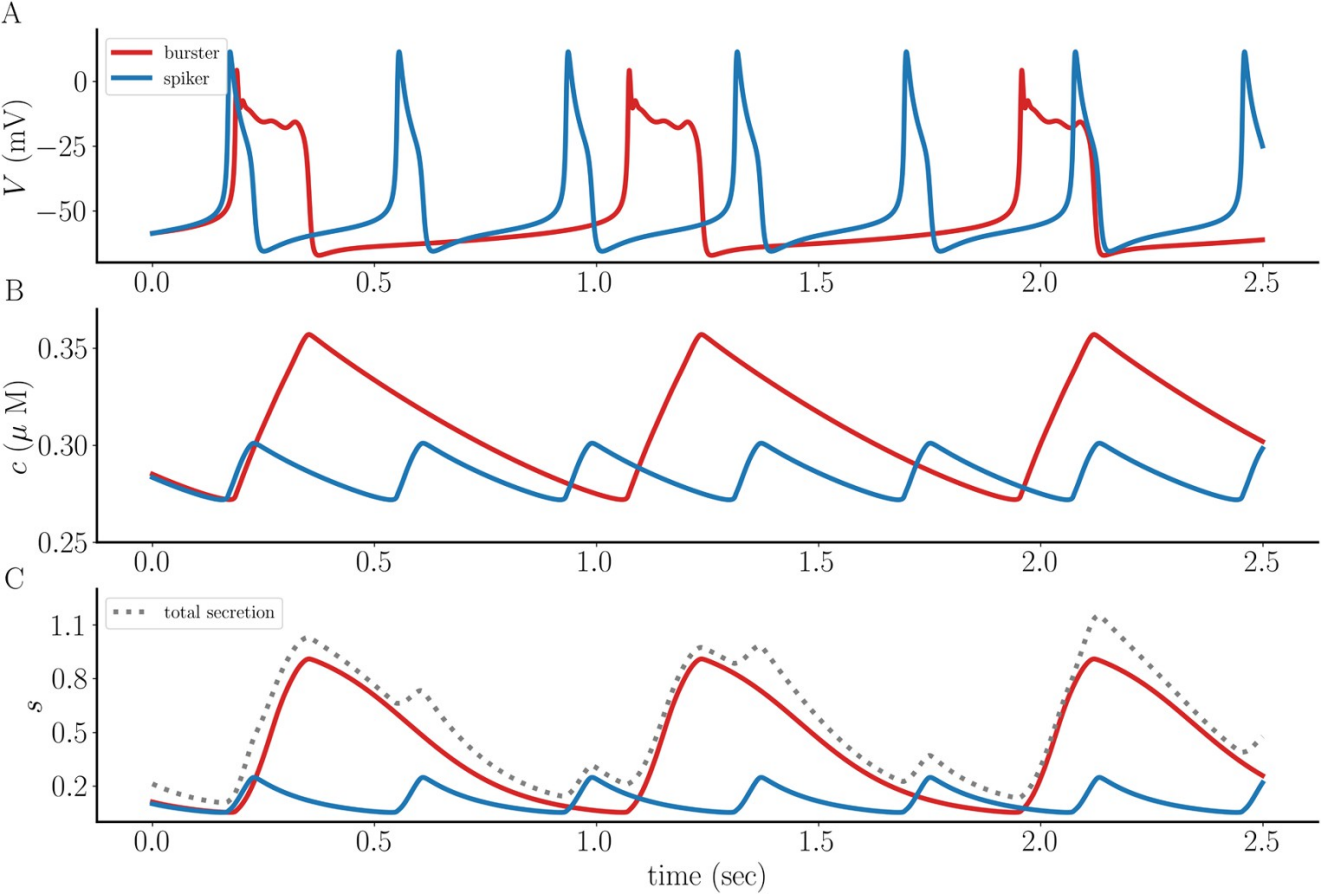
A pseudo-plateau burster (red) is weakly coupled to a tonic spiker (blue) with coupling conductance *g*_c_ = 0.005 nS. **(A)** The weak coupling is insufficient to synchronize the activity of the spiker and the burster. **(B)** The free intracellular Ca^2+^ concentration is greater for the bursting cell than the spiking cell. **(C)** The difference in *c* is amplified in the secretion variable *s*. Also shown is the sum of the *s* values from the two cells, or total secretion (dotted gray curve).

We begin with the case of weak electrical coupling (coupling conductance *g*_c_ = 0.005 nS). This is insufficient to synchronize the activity of the spiker with that of the burster, so there is persistent phase drift. This case is shown to highlight the difference in the intracellular Ca^2+^ level *c* and the secretion variable *s* between the two forms of activity. Fig 2A shows that the bursts (red) are wider than the spikes (blue), bringing in more Ca^2+^ and thus resulting in a higher intracellular free Ca^2+^ level (*c*), shown in Fig 2B. This difference is amplified in the secretion variable *s* (Fig 2C), which reflects the likely nonlinear relationship between intracellular Ca^2+^ and exocytosis due to the requirement that multiple Ca^2+^ ions must bind release sites to evoke release [17, 18]. Also shown in Fig 2C is the sum of the *s* values from the two cells (i.e., the total secretion).

**Fig 3:**
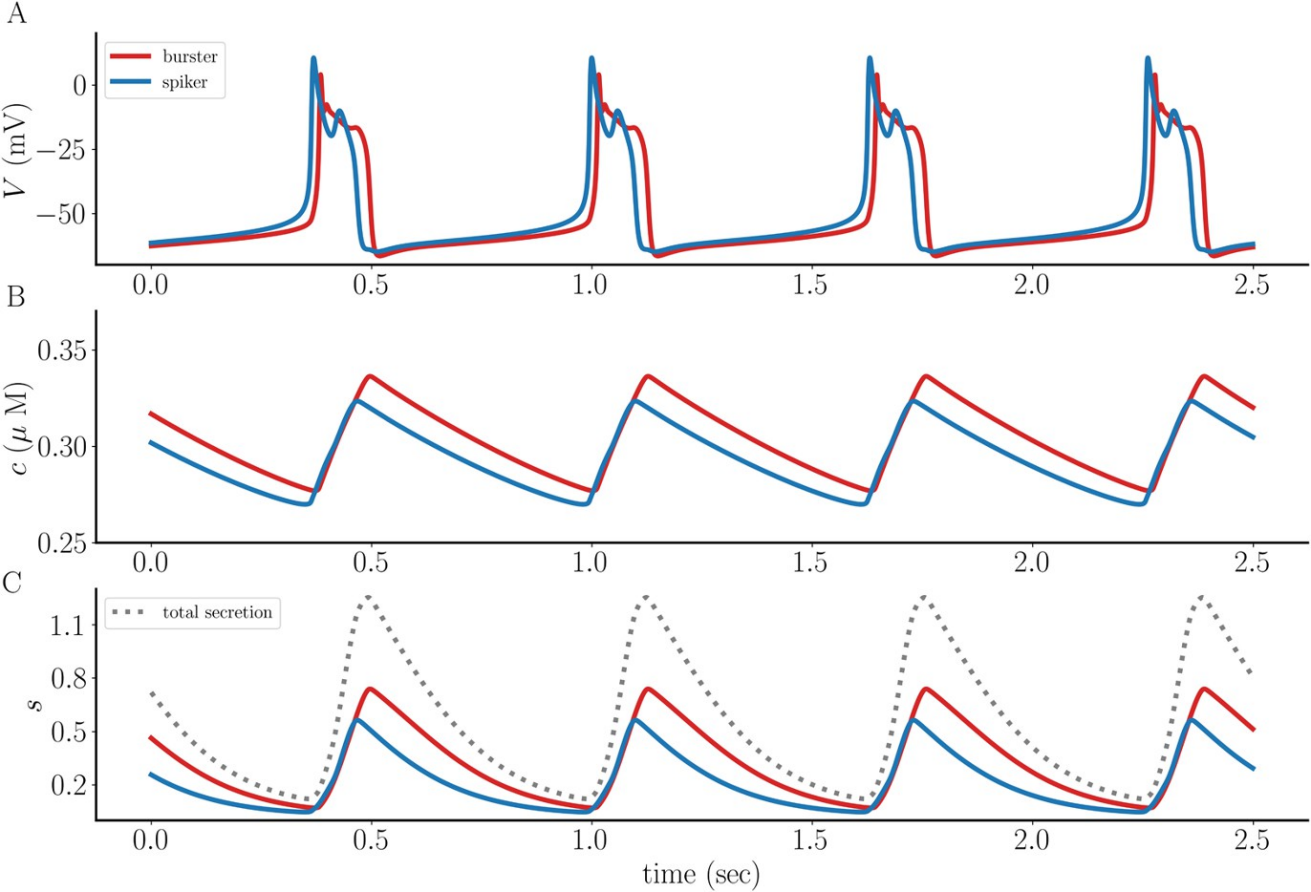
When the coupling between the intrinsic pseudo-plateau burster (red) and the intrinsic spiker (blue) is stronger (*g*_c_ = 0.05 nS) a compromise is reached that produces synchronization. **(A)** Both coupled cells now exhibit bursting, though each burst is shorter than that of the intrinsic bursting pattern of the red cell. **(B, C)** The free intracellular Ca^2+^ concentration and secretion variable are still greater for the intrinsic bursting cell, but not by much. Also shown is the sum of the *s* values from the two cells, or total secretion (dotted gray curve).

Increasing the coupling conductance by a factor of 10 (to *g*_c_ = 0.05 nS) not only synchronizes the cells, but also converts the spiker to a burster. That is, the coupling prolongs the depolarization of the spiker by converting it to a burster (blue) and thereby makes the membrane depolarization longer. It also reduces the duration of the depolarization in the intrinsic burster (red), so that there is a compromise at which the two cells synchronize (Fig 3A). The *c* and *s* variables for the intrinsic burster are still greater than those for the intrinsic spiker, but by a reduced amount, and the total secretion is greater (Fig 3B and 3C).

### Fast/slow analysis reveals how the spiker is converted to a burster

The ability of a bursting cell to convert a spiker to bursting is an important feature of the coupling that occurs frequently in the larger network simulations that are described later. Here we explain how it happens. The bursting produced by the intrinsic buster is due in large part to fast-activating K^+^ current (BK current) that facilitates pseudo-plateau bursting [19, 20]. In terms of fast/slow analysis, this current facilitates the twisting of the slow manifold that is induced by a folded node singularity [21]. If the trajectory enters this twisted region of the slow manifold, small oscillations occur in the trajectory which also prolong the depolarization. The result is a pseudo-plateau burst [15, 22]. Unlike the intrinsic burster, the intrinsic spiker lacks BK current and the associated activation variable (the spiker is a 3-dimensional model, while the burster is a 4-dimensional model). This raises the question of how bursting is produced by the intrinsic spiker when it is coupled to an intrinsic burster. Does it inherit the bursting pattern of the coupled cell, essentially mimicking the trajectory of that cell? Or does it have its own burst-producing machinery that is triggered by coupling to the intrinsic burster?

To address these questions we use a two-part approach. First, we show that the spiking cell has a twisted slow manifold that can produce the small oscillations in a pseudo-plateau burst, and then we show that this is engaged when coupled to an intrinsic burster.

The model we used for the intrinsic spiking cell has 3 variables. The voltage variable evolves on a fast time scale, while the K^+^ channel activation variable (*n*) and the intracellular Ca^2+^ concentration (*c*) evolve on slower time scales. Thus, the system can be examined using a fast/slow analysis with one fast and two slow variables (grouping *n* and *c* together as “slow”). Such an analysis was performed on a similar 1-fast, 2-slow model for pituitary electrical activity in [22, 23], so the details will not be repeated here. The key point is that for this model the slow manifolds are 2-dimensional surfaces in the 3-dimensional phase space that determine the course of the trajectory. During the lower or hyperpolarized phase of a spike or burst the trajectory moves along the lower stable manifold (a set of stable equilibria of the fast subsystem), while during the upper or depolarized phase the trajectory moves along the upper one (also a set of stable equilibria of the fast subsystem). This upper manifold can be twisted, and if the trajectory enters this twisted region then small oscillations are produced. Pseudo-plateau bursting relies on the trajectory entering this twisted region.

**Fig 4:**
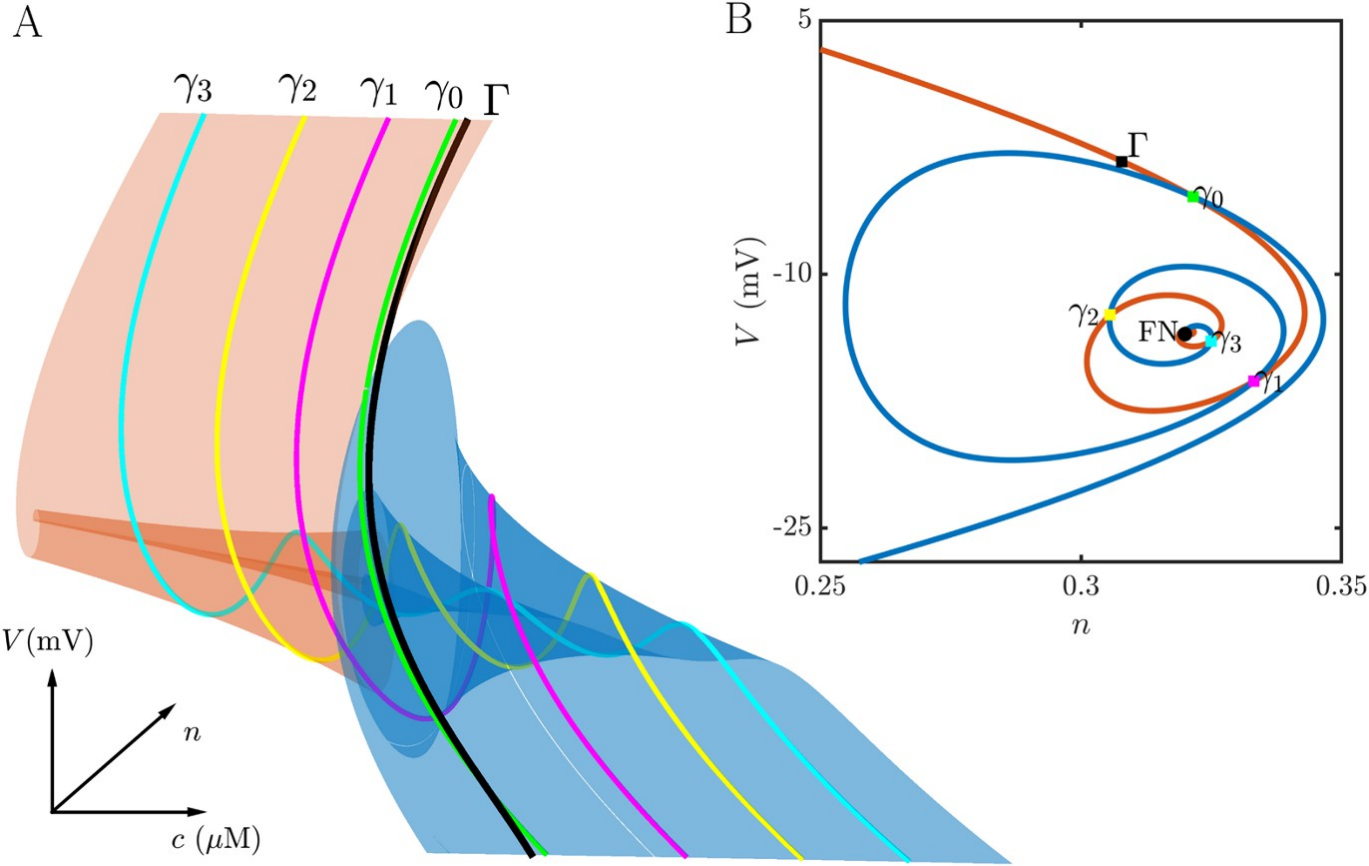
Fast/slow decomposition of the intrinsic spiking model cell. **(A)** The upper attracting slow manifold (red surface) is twisted where it meets the middle repelling manifold (blue). The primary canard (*γ*_0_) and the three secondary canards (*γ*_1_, *γ*_2_, *γ*_3_) are shown. The trajectory (black) is on the right of the primary canard, outside of the “folded funnel”, and therefore no small oscillations are produced. **(B)** A 2-dimensional slice through the twisted region of the manifolds. The red curve is the slice through the attracting manifold, while the blue curve is the slice through the repelling manifold. Intersections are slices of the canards. The twisted manifolds are organized about a folded node singularity (FN).

Fig 4 demonstrates that even in the spiking-cell model the upper slow manifold is twisted. (The methods for computing the slow manifolds and canards were developed in [24–26].) Panel A shows that the upper attracting manifold (red) intersects the middle repelling manifold (blue) along four curves, which are the primary and three secondary canards. The primary canard (*γ*_0_) has no twists, while secondary canard *γ*_*j*_ has *j* twists. (See [21] for a nice overview of canards and their properties.) If the trajectory enters the twisted region between canards *γ*_*j*−1_ and *γ*_*j*_, then it will exhibit *j* small oscillations. In the case of the tonic spiker, the segment of the spiking limit cycle that approaches the twisted region of the manifold (*Γ*) is on the right of the primary canard, so no oscillations are produced. Fig 4B shows this from a different perspective, where a slice of the twisted manifolds with fixed *c* is taken. The red curve is the slice of the attracting manifold, while the blue curve is the slice of the repelling manifold. The twists are evident here, and the intersections correspond to slices of the canard curves. The slice of the trajectory Γ is on the wrong side of the primary canard *γ*_0_ to exhibit oscillations. The organizing center of the twisted manifolds is a folded node singularity (FN), which is thoroughly described in publications such as [21, 27, 28]. In summary, then, the spiking cell model has the machinery to produce pseudo-plateau bursts, but the limit cycle trajectory does not enter the twisted region of the upper attracting slow manifold that would make the small oscillations required for pseudo-plateau bursting.

**Fig 5:**
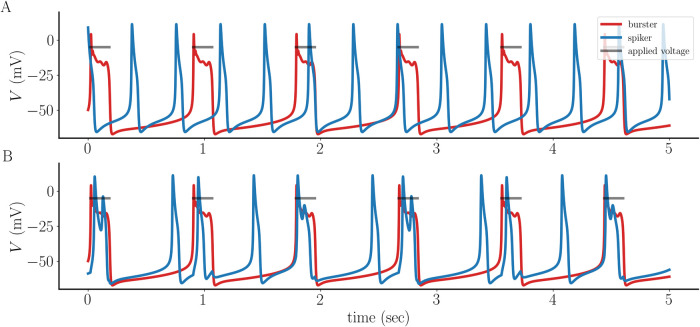
Demonstration that the full burst pattern is not needed as input to convert a spiker to a burster. **(A)** Spiking and bursting cells without coupling. The short horizontal line segments indicate how the burst voltage is approximated during the burst active phase when coupling is turned on. **(B)** With coupling (*g*_c_ = 0.05 nS), the spiker often produces bursts in synchrony with those of the intrinsic burster when it experiences constant elevated voltage during each burst, rather than the actual bursting voltage trace.

We next wished to determine whether the action of the coupled cell, a burster, causes the trajectory of the spiker to enter the twisted region of the upper attracting slow manifold and thereby become a burster itself. We note that this occurs when the intrinsic burster is in its depolarized state, and although its voltage varies during this plateau phase, we hypothesize that all that is necessary to induce bursting in the spiking cell is for the coupled cell to have pulses during which the voltage is elevated to a level similar to that of the plateau voltage. That is, rather than experiencing the full burst, the spiker need only experience an elevated constant voltage each time the burster enters an active phase. We simulate this in Fig 5. Panel A shows the behavior of the two cells without coupling, and indicates the voltage that is used as input to the spiker when coupling is turned on. As shown in panel B, applying the coupling current during the active phase of each burst of the intrinsic burster is sufficient to induce bursting in the intrinsic spiking cell. In four of the six instances that the burster produced a burst, so too did the spiker.

**Fig 6:**
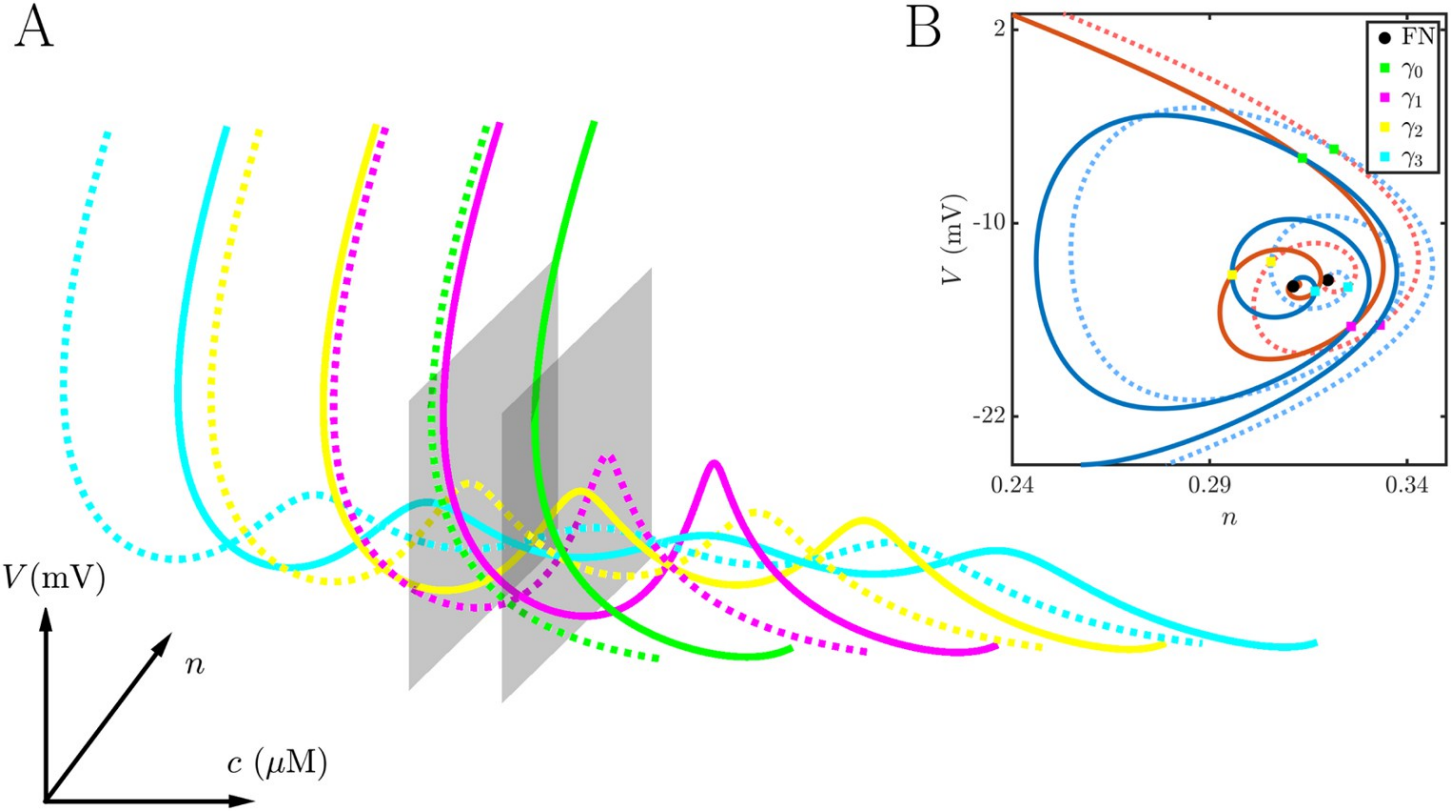
**(A)** Addition of a coupling current (with conductance *g*_c_ = 0.05 nS), in which the coupled cell is clamped at a fixed voltage as indicated in Fig 5, shifts the canards to higher *c* values. The dashed curves are the original canards without coupling current. The solid curves are the canards with coupling currents. Also shown are the planes of constant *c* that contain the folded node singularity. **(B)** Slices of the constant-*c* planes through the attracting and repelling slow manifolds with (solid) and without (dashed) the coupling current.

The effect of adding the constant voltage pulse through gap junctional coupling is to shift the canards of the spiking cell to higher values of *c*. (The maximum number of canards does not change over the range of *g*_c_ values used here, based on the ratio of largest to smallest eigenvalues of the Jacobian of the desingularized system calculated at the folded node singularity [28].) This is shown in Fig 6A. The dashed curves are the original canards, colored as in Fig 4A, and the solid curves are the new canards with the added coupling current. Each canard is right-shifted to higher *c* values. Also shown for each set of canards is the plane of constant *c* that contains the folded node singularity. This plane, and thus the folded node, is also right-shifted. Panel B shows the slice of each of these planes through the slow manifolds, with the slices through the canards indicated. It is evident that the coupling current shifted the entire slow manifolds.

**Fig 7:**
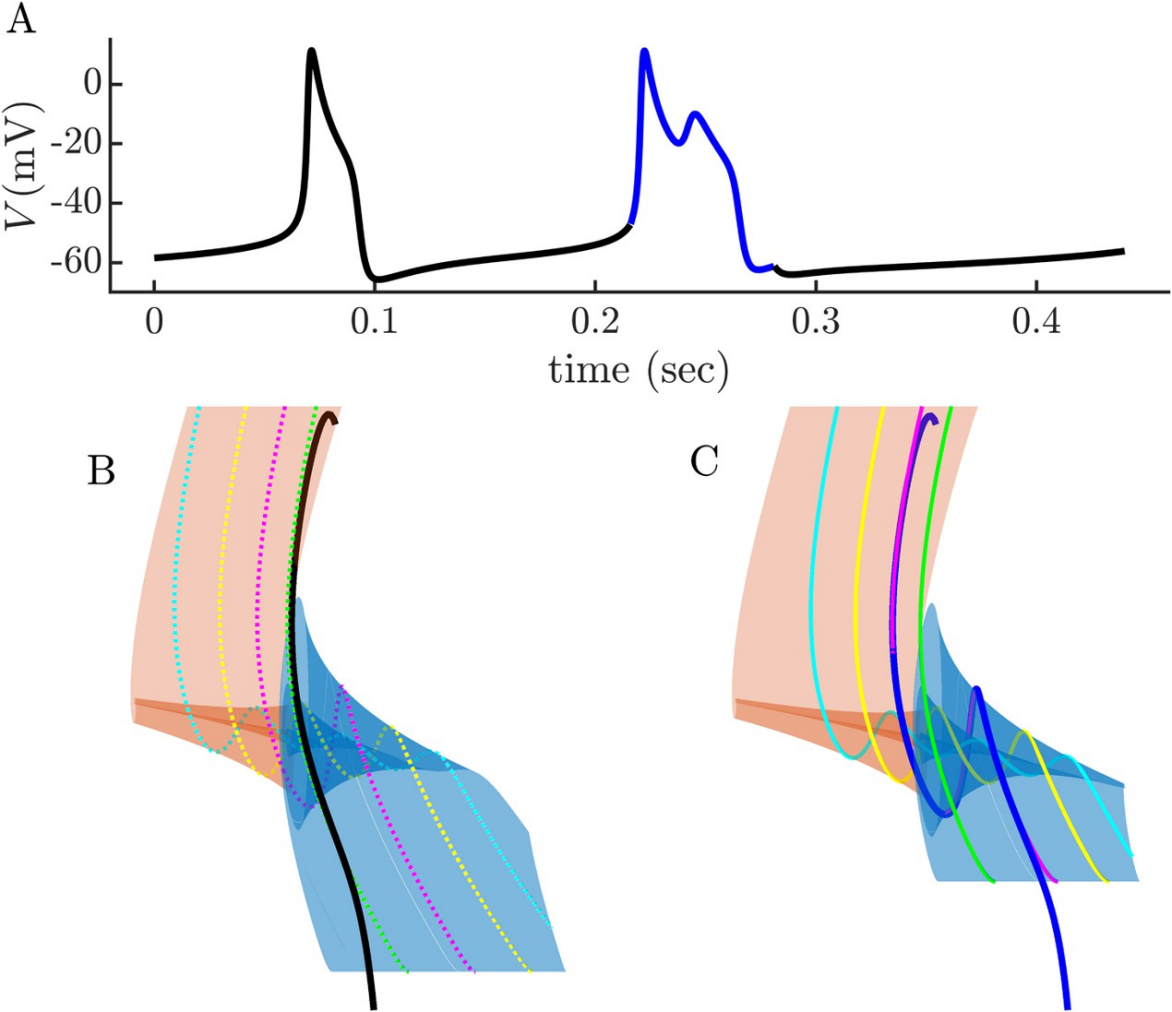
**(A)** A constant coupling current (with conductance *g*_c_ = 0.05 nS) is added to an intrinsic spiker during the second of two events in the time series (blue). This converts the spike to a 2-spike burst. **(B)** Slow manifolds, canards, and spike trajectory when no coupling current is added to the intrinsic spiker. The trajectory (black) enters the twisted region on the right of *γ*_0_, generating a single spike. **(C)** Slow manifolds, canards, and burst trajectory of the intrinsic spiker with coupling current added. The trajectory (blue) enters the twisted region near *γ*_1_, generating a burst with two spikes.

Fig 7A shows a short time trace of the intrinsic spiker in which the coupling current is applied only during the second event (in blue), not the first. Underneath, in panels B and C, shows the twisted slow manifolds with canards for both events. Without the coupling current, the trajectory (black) passes on the right of the primary canard (green), so there are no small oscillations produced. During the second event, a 2-spike burst, the trajectory enters the twisted region between the canards *γ*_0_ and *γ*_1_, so one small oscillation is generated, which is the second spike in the 2-spike burst.

**Fig 8:**
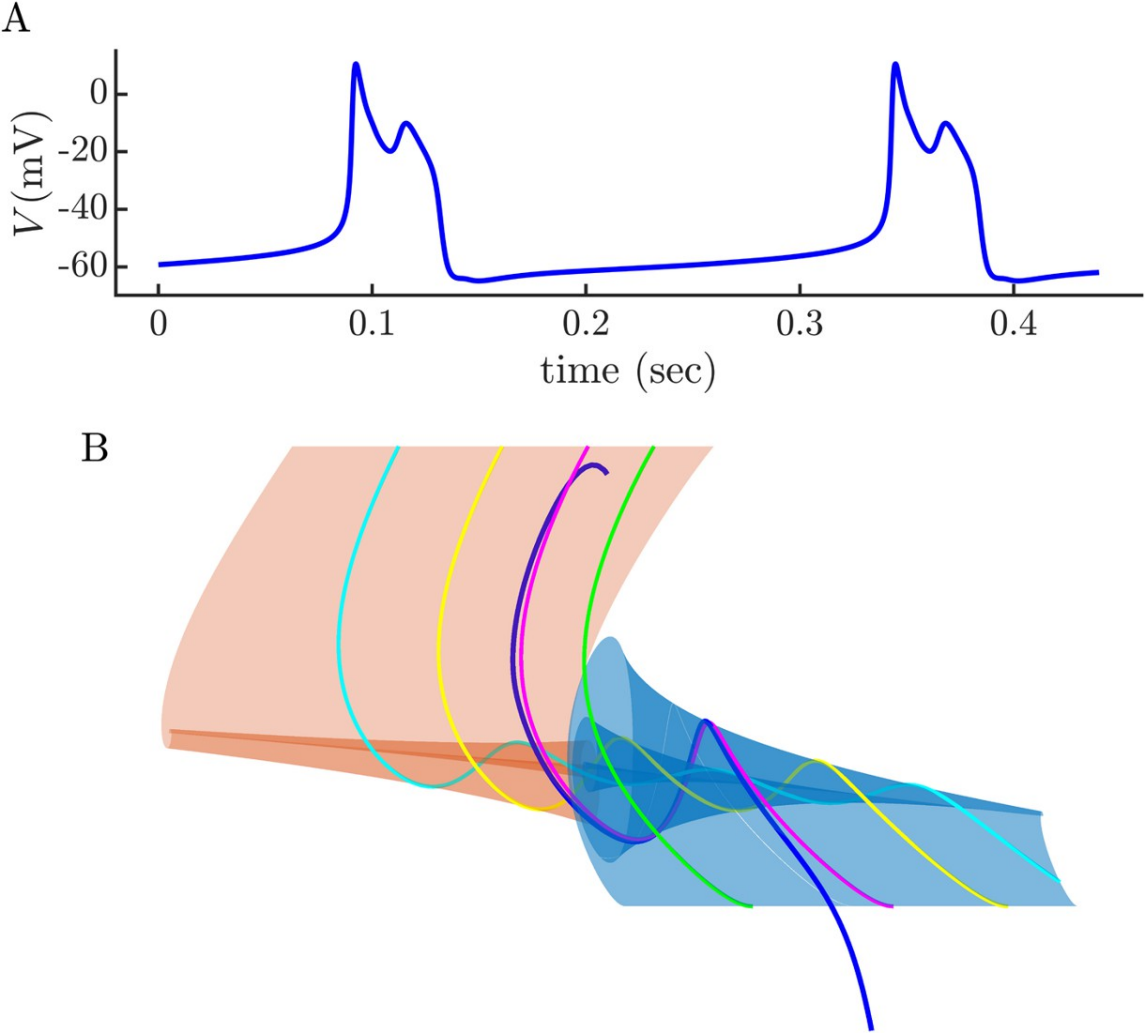
**(A)** Bursting produced when the intrinsic spiker is coupled (with *g*_c_ = 0.05 nS) to an intrinsic burster, producing 2-spike bursts. **(B)** A segment of the burst limit cycle is overlaid on the slow manifolds generated with a constant coupling current. The trajectory (blue) enters the twisted region near canard *γ*_1_, just as it did in Fig 7.

The structures shown above were computed with a fixed value of *V* in the coupled cell applied during its active phase. How does the trajectory of the intrinsic spiker, coupled to a burster with time-varying *V*, fall on the slow manifolds constructed above? This is shown in Fig 8. Two-spike bursting is produced, and the trajectory enters the twisted region in a location that is quite similar to where it entered with a fixed coupling current, indicating that the small time variation of *V* in the coupled cell is not important for generating bursting in the intrinsic spiker.

In summary, the analysis demonstrates that the spiker has the necessary machinery to burst, and that coupling to a cell with pulses of elevated voltage is all that is needed to engage that machinery and produce a burst.

## Studies of small networks illustrate the complexity of network dynamics

**Fig 9:**
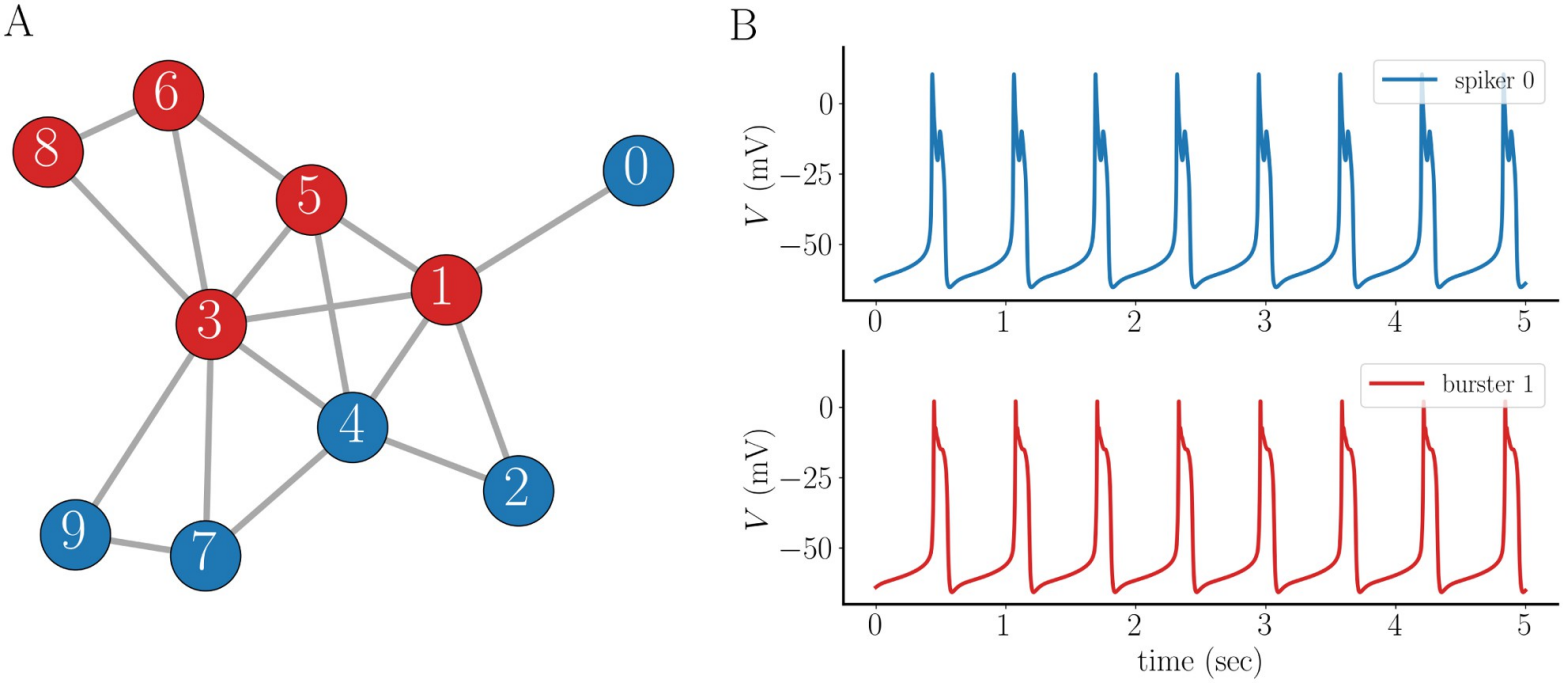
Dynamics of a small random network with electrical coupling conductance *g*_c_ = 0.05 nS at each edge. **(A)** Each red node in the network is an intrinsic burster, and each blue node is an intrinsic spiker. **(B)** Voltage traces of cells 0 and 1 in the network. Both produce uniform bursting, as do all other cells in the network.

The last section demonstrated that, with sufficiently strong electrical coupling, intrinsic bursters can effectively convert intrinsic spikers to bursters. How does this play out in a larger network? To address this question, we construct a random network of 10 cells using the method described in Section 2. This network is shown in Fig 9A. The nodes are color-coded according to their intrinsic spiking (blue) or bursting (red) behavior. In this example, the two populations are arranged in mostly homogeneous clusters. The result of the network interactions is that all spikers are converted to bursters and all burst patterns are shorter than without coupling (Fig 9B), as in Fig 3A. Also, all cells are synchronized.

**Fig 10:**
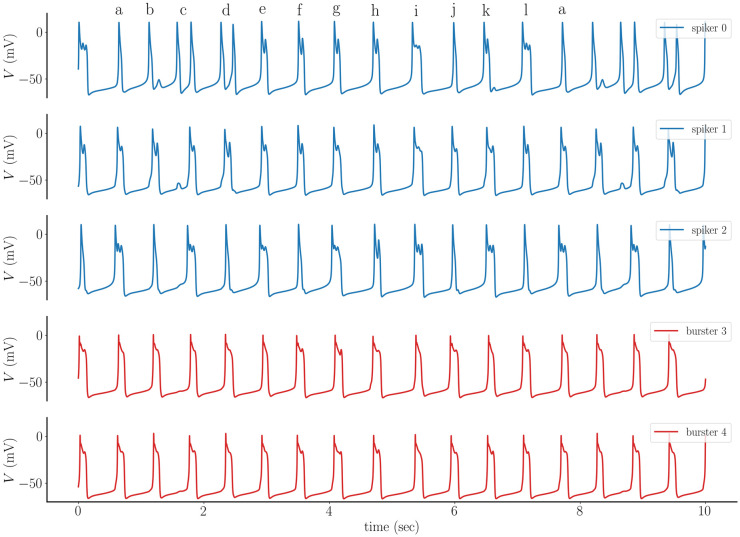
A small change in the cell properties can have a large impact on the network dynamics. Voltage time courses from five cells in the network of Fig 9A in which the intrinsic properties of cells 1 and 4 are switched. The first three cells shown are intrinsic spikers, while the last two are intrinsic bursters. The network dynamics are complex, with a mix of spikes and bursts. Labels over the top panel demonstrate that the pattern of activity forms a period-12 cycle. Coupling conductance is *g*_c_ = 0.05 nS.

In the next example, the intrinsic properties of two adjacent cells are flipped; node 1 is changed from an intrinsic burster to an intrinsic spiker, and node 4 is changed from an intrinsic spiker to an intrinsic burster. With this change, the bursting population remains clustered together, while the spiker population is split into two clusters. The impact on the network dynamics is profound. Cell 0, an intrinsic spiker, now exhibits a mix of spikes, spike doublets, two-spike bursts, and longer bursts (top panel, Fig 10). Cell number 1, an intrinsic spiker, becomes a burster, but the bursts are not uniform, showing differences in duration from one to the other. In fact, it is conversion of cell 1 to bursting that in turn converts cell 0 to bursting. Cell 2, also an intrinsic spiker, exhibits a mostly period-2-type pattern with alternating spikes and bursts. There are, however, instances of sequences of several bursts. Finally, cell 3, an intrinsic burster, is still a burster within the coupled network, but as in cell 1 the bursts are non-uniform. It is clear, then, that this small change to the cell properties within the network produced a very large, and unpredictable, change in the network dynamics.

The voltage time courses shown in Fig 10 appear to be chaotic, but are they? It turns out that the activity pattern is actually a period-12 sequence. This can be seen by examining the voltage time course of cell 0. The sequence contains two spikes (labels **a** and **b**), followed by two spike doublets (**c** and **d**), followed by four 2-spike bursts (**e**, **f**, **g**, and **h**), a longer burst (**i**), a spike (**j**), a 2-spike burst (**k**) and a longer burst (**l**). This pattern repeats periodically.

## Strategies for maximizing secretion

The last examples show that the placement of the spikers and bursters in a network can have profound effects on the network dynamics. Converting a spiker to a burster increases secretion from that cell, both in the model and in actual pituitary cells. However, coupling a burster to a spiker tends to decrease the duration of the burster, which would reduce secretion from that cell. A natural question is how could intrinsic bursters be placed within a network to maximize secretion across the network?

**Fig 11:**
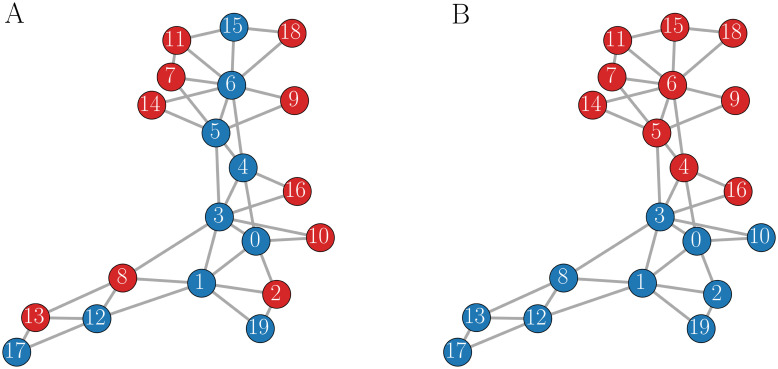
Two 20-cell networks in which 50% of the cells are intrinsic bursters (red) and 50% are intrinsic spikers (blue). **(A)** The bursters are distributed according to an offensive strategy in which they contact most of the spikers, yielding a low burster homophily value of Γ_*b*_ = 0.12. **(B)** The bursters are clustered together, exhibiting a defensive strategy with homophily value Γ_*b*_ = 0.89.

To address this question, and thereby establish a strategy for a good placement of intrinsic bursters in the network to enhance secretion, we considered somewhat larger networks of 20 cells, coupled together randomly as described in Section 2. As a measure of the extent to which intrinsic bursters are clumped together in the network we define the “burster homophily” *γ*_b_ as the fraction of neighbors of an intrinsic burster that are also intrinsic bursters, and Γ_b_ as the average of these values across all intrinsic bursters in the network. (We also define a similar parameter, *γ*_s_, as the fraction of neighbors of an intrinsic spiker that are intrinsic bursters, and Γ_s_ as the average of *γ*_s_ over the intrinsic spiker population.) For example, in Fig 11A where 50% of the cells are intrinsic bursters, *γ*_b_ for cell 2 is 0, while *γ*_b_ for cell 13 is 1/3. The average over the network, Γ_b_, is 0.12. In contrast, Γ_b_ is much larger, 0.89, for the network with different placement of intrinsic bursters shown in Fig 11B, indicating that in this network the intrinsic bursters are much more clustered together. The distribution of cells in the first network can then be seen as playing an “offensive strategy”, spreading out the bursters so as to contact as many spikers as possible to possibly convert them. The second network exemplifies a “defensive strategy” in which the bursters are clumped together, protecting them from the burst shortening that can happen when coupled to spikers.

**Fig 12:**
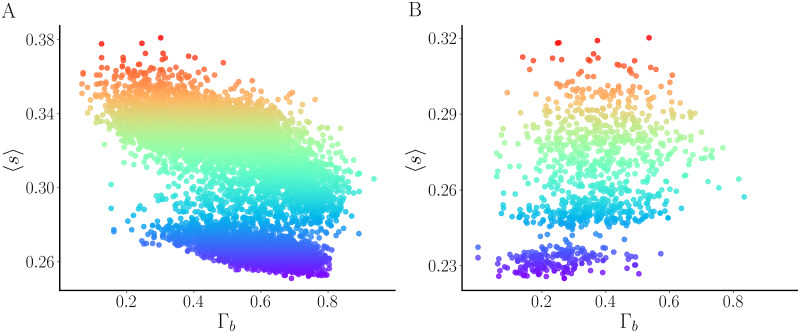
Scatter plots showing average secretion 〈*s*〉 as a function of the average burster homophily Γ_b_ for 20-cell networks with coupling parameter *g*_c_ = 0.05 nS. **(A)** 10,000 simulations were performed with the 50% of cells that are intrinsic bursters distributed in different ways in the network of Fig 11. In general, networks with lower values of Γ_b_, an offensive strategy, produce higher secretion. **(B)** 1000 simulations were performed with the 25% of cells that are intrinsic bursters distributed in different ways. The secretion produced by networks with higher values of Γ_b_ is generally greater than that produced by networks with lower Γ_b_, indicating that a defensive strategy is better.

Which strategy is best for maximizing secretion, an offensive or a defensive strategy? To address this question, we took the network structure from Fig 9 and distributed the 50% of cells that are intrinsic bursters in 10,000 different ways. For each of these networks, we calculated Γ_b_ and the average value of the secretion variable *s* across the network (〈*s*〉) and over a time span of 10 seconds. Both of these values are plotted in the scatter plot of Fig 12, where each point is colored according to the size of 〈*s*〉. Panel A shows that the highest secretion values occur for the lower values of Γ_b_, i.e., when the bursters are organized according to an offensive strategy. At the lowest values of Γ_b_ (below Γ_*b*_ = 0.25), the vast majority of the networks yield high secretion. In contrast, at the highest values of Γ_b_ (above Γ_*b*_ = 0.6), the vast majority produce either low or moderate secretion values. Thus, the offensive strategy is generally better than the defensive strategy at producing high secretion when 50% of the cells in the network are intrinsic bursters.

While an offensive strategy may be best when 50% of the cells are intrinsic bursters, it is quite possible that the best strategy could change when there are fewer intrinsic bursters. We examined this by again distributing intrinsic bursters randomly throughout the network, but this time only 25% of the cells are intrinsic bursters. Because there are fewer cells to distribute, we performed fewer simulations (1000 rather than 10,000), but the pattern is clear. As shown in Fig 12B, networks with lower burster homophily often have lower secretion than those with higher homophily values. Thus, when the fraction of cells in the network that are intrinsic bursters is 25%, it is best to take a defensive strategy, with higher burster homophily values.

**Fig 13:**
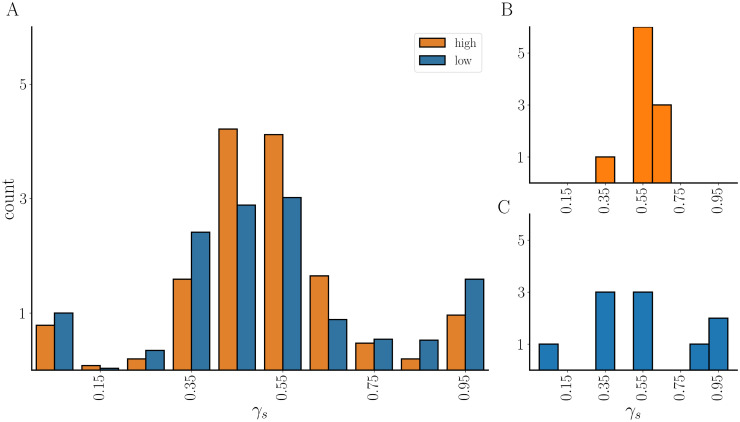
**(A)** Distribution of *γ*_s_ over populations of low-secreting networks (blue) and high-secreting networks (orange) all with the same values of Γ_b_ (0.55) and Γ_s_ (0.52). The low-secreting population has a wider distribution. **(B)** Distributions of *γ*_s_ for a high-secreting and **(C)** a low-secreting network.

One thing that stands out from the scatter plots of Fig 12 is that there are two clusters of points in each plot. For the same value of Γ_b_ there are networks in the lower cluster that secrete less and networks in the upper cluster that secrete more. What is the difference between the distribution of spikers and bursters in these low-secreting and high-secreting networks? To find out, we examined a population with 112 networks, all of which had the same value of Γ_b_ (the fraction of neighbors that are bursters, averaged over all bursters in the network) and Γ_s_ (the fraction of neighbors that are bursters, averaged over all spikers in the network). We then constructed a histogram of *γ*_s_ values by binning the *γ*_s_ values for all networks in the low-secreting subpopulation, since this reports the fraction of a spiker’s neighbors that are bursters and can potentially transform the cell to a bursting state (although even a spiking neighbor can convert a cell to bursting, if that neighbor is converted to bursting). This histogram is shown as blue bars in Fig 13A and a histogram for a single low-secreting network is shown as blue bars in panel C. Similar histograms are constructed for the high-secreting networks, and shown as orange bars (panels A and B). From the population histogram, it appears that the distribution of *γ*_s_ for low-secreting networks is wider than that of the high-secreting networks. This is also shown for two individual networks in panels B and C; the low secreting network is more spread out than the high-secreting one. A spiking cell with a large *γ*_s_ would likely be converted to a burster, increasing secretion and opening up the possibility that it can then convert its spiking neighbors to bursters. A wider distribution of *γ*_s_ across the subpopulation of low secreting networks likely means that fewer cells were exposed to a large fraction of bursting neighbors. For this reason, they were not converted to bursters and the secretion was low.

**Fig 14:**
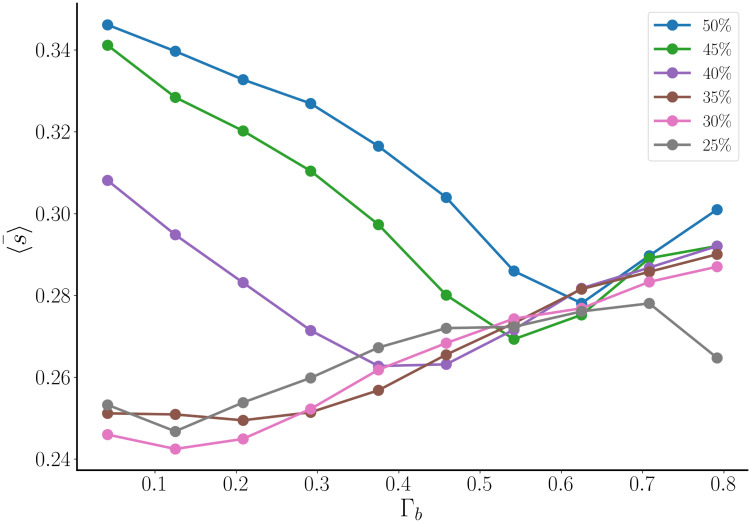
Secretion data from Fig 12 is binned and averaged, yielding the average over a bin $\bar{\langle s\rangle }$. This is done for six different values of the intrinsic burster fraction. For low intrinsic burster fraction, secretion is maximized when Γ_b_ is large. For a high intrinsic burster fraction, secretion is maximized when Γ_b_ is low.

The simulations described in Fig 12 suggest that a defensive strategy is best for secretion when Γ_b_ is low, and an offensive strategy is best when Γ_b_ is high. Is this really true? To investigate further, we quantified the scatter plot information by binning the data with 10 bins of equal size for Γ_*b*_ ∈ [0, 1]. The data points falling into each bin were averaged, yielding a mean value $\bar{\langle s\rangle }$. This was done for networks with different fractions of intrinsic bursters, from 25% up to 50% in equal increments of 5%. The result is shown in Fig 14. With a low fraction of intrinsic bursters (25–35%), the average secretion is highest when Γ_b_ is highest. That is, when the intrinsic bursters are clustered together and playing a defensive strategy. With a high fraction of intrinsic bursters (40–50%), the opposite is true. The secretion is maximized when Γ_b_ is low and therefore the intrinsic bursters are spread out and play an offensive strategy. It is curious that for the higher intrinsic burster fraction the minimum secretion occurs at an intermediate value of Γ_b_, rather than the largest value of Γ_b_. This indicates that the worst strategy is not a defensive one, but one that is a compromise between offensive and defensive.

## Discussion

We have demonstrated the large impact that gap junctional coupling can have on the electrical activity of pituitary cells such as lactotrophs, somatotrophs, and corticotrophs that, as single cells, can exhibit spiking activity or pseudo-plateau bursting. Because the coupling tends to convert the spikers to bursters, the mathematical model predicts that the fraction of bursting cells in the coupled network will be considerably higher than what would occur in dispersed cells. The model also predicts that the way in which the intrinsic bursters are distributed in the network is very important for the level of hormone secretion. If the fraction of cells in the network that are intrinsic bursters is low, then there is in general more hormone secretion if the bursters are clustered together with low exposure to intrinsic spikers. If the fraction of cells that are intrinsic bursters is higher, then secretion is generally increased if the bursters are dispersed throughout the network with high exposure to intrinsic spiking cells.

There have been quite a few mathematical studies of bursting cells coupled through gap junctions. These have been in relation to pancreatic islets, where the insulin-secreting *β* cells are coupled to other nearby *β* cells. Some of these studies were mathematical in nature and focused on two coupled bursting *β* cells [29–31], or were computational in nature focusing on larger networks of bursting *β* cells [32–35]. Several modeling studies of *β* cells examined coupling between spiking and bursting cells [30, 36, 37], and showed that bursters can convert spikers into bursters, particularly with the help of noise. However, the type of bursting produced by these models, “square-wave” or “plateau” bursting, is different from the pseudo-plateau bursting produced by models of pituitary lactotrophs, somatotrophs, and corticotrophs. The most obvious difference is that the plateau bursts are characterized by large spikes collected into episodes, while pseudo-plateau bursts consist of small voltage oscillations riding on a plateau. In terms of the underlying dynamical structures that give rise to the bursting, they are very different. Plateau bursting involves bistability of spiking and silent states in the fast subsystem of variables [38–40], while pseudo-plateau bursting does not [41], but instead, the small oscillations are due to canards [22, 42]. For these reasons, the results from prior studies should not be expected to carry over to pituitary cell networks. The current study is a follow-up to a recent one from our group, which considered weak coupling of identical pituitary bursters and demonstrated the difficulty in predicting the functional network defined by cell synchronization from the structural network of cell coupling [10].

Besides the type of bursting produced by the cells, another difference between pituitary networks and pancreatic islets is that the islets are roughly spherical, and in the mouse (the focus of most modeling studies) the *β* cells form the core of the sphere [43]. Because of this structure, the number of neighbors of each *β* cell should be relatively uniform. In contrast, pituitary networks have no clear structure [4–7], which allows for greater variability in the number of neighbors of any cell. There can be hubs, for example, with most cells having few neighbors, but some cells having many. This non-uniformity allows for much greater flexibility in the network dynamics that result from various placements of spikers and bursters within the network. For example, cells 1 and 4 in Fig 9A both connect to 5 other cells, so have a high degree relative to other cells in the network. Flipping the intrinsic nature of these cells between spiking and bursting had a big impact on the network dynamics (Fig 10). One might think that flippling the intrinsic properties of cells with low degree, for example cells 8 and 9 in Fig 9A, might have a smaller effect. However, we also found that flipping these cells had a large effect on the network dynamics. This points out the extreme difficulty in predicting the effect of small changes in the network on the dynamics of the network. We found similar sensitivity in our earlier work with networks of weakly coupled pituitary bursters [10].

We limited the heterogeneity of the cells within the network to just two types, based upon the value of a single parameter for the conductance of BK-type channels. This parameter was chosen since it has been shown that addition of BK current with dynamic clamp [44] to a pituitary cell with BK channels blocked can convert the cell from a spiker to a burster [19, 45]. There is certainly more heterogeneity among pituitary cells than this, even among a single type of cell, so cells within a network of lactotrophs, for example, would be expected to exhibit a great deal more heterogeneity in their intrinsic electrical activity than we have included in our simulations. It is also possible to incorporate heterogeneity in the strength of coupling, so that some links between two cells are significantly stronger than others. Biologically, this would mean a greater number of gap junctions connecting some cell pairs than others. These sources of heterogeneity just makes the complicated network dynamics even more complicated. Experimental studies of the effects of gap junctional coupling on pituitary cell activity, as we have done here mathematically, would be hard to perform. Perhaps with application of tools such as the dynamic clamp some of the predictions made in this study will be tested experimentally in the future.
